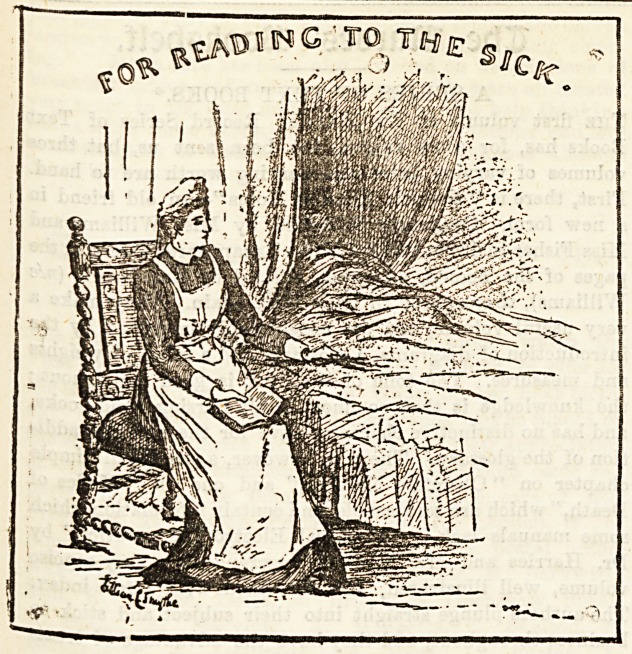# The Hospital Nursing Supplement

**Published:** 1891-08-22

**Authors:** 


### "The Hospital" Nursing Mirror.

Being the Extra Nursing Supplement of "The Hospital" Newspaper.

Contributions for this Supplement should be addressed to the Editor, The Hospital, 140, Strand, London, W.C., and should have the word "Nursing" plainly written in left-hand top corner of the envelope.

### En Passant.

WEYMOUTH ROYAL HOSPITAL.—The Matron of this institution makes a special appeal for assistance in aid of the institution, which is greatly in need of funds, and it is open night and day for accidents of all kinds. Any amount sent, however small, will at once be acknowledged by her. The hospital is not endowed, and is supported entirely by voluntary contributions.

SHEFFIELD NURSES' HOME.—The annual meeting of the Sheffield Nurses' Home and Training Institution was recently held at the new Home, Glossop Road. The institution has just been removed to more commodious and convenient premises adjoining the previous establishment, and this was the first official gathering under the new conditions. The Ven. Archdeacon Blakeney presided. The Committee, in presenting their twentieth annual report, congratulated the subscribers on the improved financial condition of the institution. The earnings of the nurses had during the past year increased by £221 12s. 1d. The Committee expressed their infinite sorrow at the loss during the year of their colleague, Mr. Jonathan Barber, whose services as Honorary Secretary to the institution could never be forgotten. Mr. Jonathan Barber first conceived the idea of a Nurses' Home for Sheffield. There was nothing of the kind in the town or neighbourhood then, and when anyone wanted a nurse they had to go to Lincoln or some other town to obtain one. At the termination of the proceedings the Ven. Archdeacon presented to Miss Armstrong, the Lady Superintendent, a kettle and stand, on behalf of the nurses under her charge.

NURSES AND DOCTORS.—There is always something satisfactory when one's power as an adversary is recognized, even by the best of one's friends. Nurses can, therefore, take it as somewhat of a compliment that the last meeting of the Medical Congress at Bournemouth devoted some time to considering the best method of protecting the medical, against the encroachments of the nursing, profession, and a very animated debate arose on the subject. Dr. T. Groves moved the following resolution: "That it is desirable that nurses, medical, surgical, obstetric, &c., shall in the performance of their duties be entirely under the control of the medical profession, and that the Council be requested to consider and report as to the best means to attain this end." He said that this was a question which would have to be grappled with sooner or later by the medical profession. There had grown up alongside of their profession a power and influence which was ever growing, whose interests were becoming exceedingly strong. His opinion was, and it was an opinion held by many others, that unless the medical profession managed to control their influence it would very quickly control the medical profession. It seemed to him (Dr. Groves) that such a condition of things ought not to be. He was called recently to a case where most injudicious feeding was being carried on. He (Dr. Groves) asked the medical man if it was done with his consent. "No," he said, "but I dare not try to control the nurse; she would at once do all she knew to prejudice me." Dr. M. F. Brook (Fareham) seconded the motion, as put by Dr. Groves, and Dr. H. A. Lawton (Poole) said that the picture Dr. Groves had painted was no fanciful one. He often found the district nurses interfering in treatment without the slightest permission or consent on the part of the medical man. Some of the medical men present entirely disagreed with the remarks made in disparagement of nurses. Surgeon-Major Ince remarked that the attempt to control nurses was as likely to succeed as would the experiment of trying to send the sea from these shores to the shores of Cherbourg. After some remarks by Mr. Ernest Hart, who deprecated the vague terms of the resolution, and said it ought not to be carried, the President put it to the meeting, and on the show of hands declared it to be adopted. We shall await the report of the action taken on the resolution by the Council of the British Medical Association with interest.

HOSPITAL NURSES.—A correspondent, signing himself "A Patient," writes to a Liverpool paper concerning hospital nurses in very eulogistic terms. Praise of the nursing staff from the inmates of hospitals is not quite so common as the public might suppose. The recipients of their care on the whole take all attention that comes in their way as a matter of course, and show very little consideration towards nurses. However weary they may be, words, such as used by the inmate of the Liverpool Hospital, are not to be despised. He says: Doctors are the mainsprings of our great hospitals, but what would the patients confined therein do without the kindness and undying energies of the nurses attached to those institutions? I have had the experience of a sojourn in our Royal Infirmary for a considerable time, and whilst I was obliged to be there I can honestly say I have never witnessed such scenes of love, patience, and humility on the part of any person as I did on the part of the nurses attached to that most noble of institutions. From early dawn till dark of night they have one continuous round of duties, and it must be clearly understood that their duties are by no means pleasant. They have to contend with all kinds of individuals, pleasant and otherwise, and I can assure you the pleasant are few and far between. No matter how hard to please the patient may be, it does not ruffle the temper of the nurse; on the contrary, it seems to afford the nurse more opportunity to show her love and kindness to the suffering inmates confided to her care.

QUEEN VICTORIA'S JUBILEE INSTITUTE FOR NURSES.—Since the establishment of this Institute it has extended its sphere of usefulness to country districts, as well as town populations, and the Rural Nursing Association having cast in its lot with the Institute has helped to form what is now a very important nursing movement. Nineteen districts now belong to the Rural District Branch, and it is believed the work will progress most rapidly, when the supply of trained nurses at the disposition of the Institute becomes greater. Insufficiency in this direction is preventing many localities from being undertaken. It is a matter to be regretted that the Institute makes so great a point of employing mainly the comparatively few nurses trained especially for them. A much larger amount of useful work could be undertaken were this point not so strictly adhered to. The Central Committee recognising the difficulty in the organisation of nursing in country districts, supplies local committees and Superintendents with well tried rules and advice, besides recommending fully qualified nurses to them. Papers are issued by the Institute for the benefit of workers, and Secretaries are ready to visit localities where their assistance may be of use. In order to pave the way to the establishing of a district nurse the Central Committee frequently arranges a series of lectures in villages on nursing subjects. This arrangement has proved very satisfactory where it has been tried. The Association is excellently organised, and is deserving of increased interest and support.

### Lectures on Surgical Ward Work and Nursing.

By Alexander Miles, M.B. (Edin.), C.M., F.R.C.S.E.

XXXIII.—BANDAGES FOR THE HEAD.

In applying bandages to the head you will do well to make use of the various prominences on the skull as fixing points to prevent the bandage slipping. The chief projections useful in this way are (1) *the external occipital protuberance*, which is situated at the back of the head close to where the head joins the neck. It is always a well-marked elevation, and a bandage placed below it will be effectually prevented from slipping upwards; (2) *the parietal eminences* which are placed right above the ears on the side of the head. They vary greatly in size in different persons, but are always sufficiently prominent to fix a bandage placed between them and the upper edge of the ear, and prevent it slipping upwards; (3) the *ear* effectually prevents the bandage slipping downwards; (4) *the superciliary ridges*, or upper margins of the orbit on which the eyebrows are placed, prevent the downward displacement of the bandage passing round the forehead, while (5) *the frontal eminences* or prominences of the brow equally prevent its upward displacement.

The *divergent spica* is the type of bandage chosen for covering in the head, and in applying it three sets of turns are made: (1) A *horizontal* set, which pass *round* the head above the level of the ears, being fixed in position by the anatomical points just mentioned, behind by going below the occipital protuberance, and in front between the ridges of eyebrows, and the prominences of the forehead. (2) A *coronal* set, which travel across the crown of the head from side to side, and under the chin. These turns sometimes pass in front of the ears, sometimes behind them, depending on the part of the head which is being covered in. If the front part, then go behind the ears so that the turns will be prevented from slipping forward; if the back part, then of course in front of the ears. (3) To fix the horizontal and coronal turns a single loop is made from behind forwards, and to it the others are pinned. This turn is not absolutely necessary, but it makes the bandage look neater, and if properly applied, gives additional security. At the various crossings of the different turns safety pins are inserted, or they may be stitched together with needle and thread.

To Bandage the Fore Part of the Head.—Grasp the loose tail of the bandage in the left hand, leaving about a foot free. From the left ear carry a turn *horizontally* round the head, and on reaching the starting point let the head of the bandage pass *under* the loose tail. The next turn is to go *vertically* round the head, *i.e.*, across the crown and under the chin. These two turns fix the bandage. Now begin the divergent spica by making a turn pass across the middle of the front part mapped out, and from this let succeeding turns diverge till all is covered in. The loose tail is used as a fixed point round which all the succeeding turns are twisted. It will be seen that only one turn goes under the chin, all the others going horizontally round the head. The antero-posterior turn is now made by carrying a turn from the occipital protuberance forward to the root of the nose, and to this turn all the others are pinned or stitched.

To Bandage the Posterior part of the Head.—Again the divergent spica is selected as the type of bandage, but on account of the shape of the head greater difficulty is experienced in preventing the turns slipping. Turns must be made round the chin or forehead as is found necessary to give security to the bandage.

The *Capeline or Double-headed Roller* is employed to cover in the whole of the head. It is a very secure bandage, but has the great disadvantage of heating the patient too much, and on this account is seldom used. To apply it, begin by sewing together the tails of two ordinary six-yard bandages. As one of the bandages should be somewhat longer than the other, roll about a yard or a yard and a-half of one bandage on to the other. The shorter of the two bandages is to repeatedly traverse the head antero-posteriorly, while the longer goes round and round the head fixing in the antero-posterior turns. Stand behind the patient, who should be seated on a chair, and holding the larger roller in the left hand and the smaller in the right, begin by placing the bandage across the forehead, just above the root of the nose; carry both rolls to the back of the head and then change hands, letting the smaller one pass under the larger, and so be fixed by it. Here the first antero-posterior turn is made by passing from the occiput forward across the centre of the head to the root of the nose, where the circular turn catches it in. From this mesial turn the succeeding ones diverge first on one side and then on the other, being fixed always by the circular turns till all the head is covered in. By diverging slowly and coming well down in front and behind a very secure bandage will be applied. Pins or stitches may be inserted here and there to add to the security.

The *Four-tailed bandage* for fracture of the lower jaw consists of a piece of bandage about a yard long split longitudinally, save for a short distance near the middle. In the centre of the unsplit portion a small diamond-shaped slit is made, and into it the tip of the chin is placed. The two anterior tails are carried backwards and fixed above the occipital protuberance, while the posterior ones are tied over the crown of the head. The ends of these two turns are now tied together to prevent slipping. If properly applied the patient should have difficulty in opening his mouth.

Handkerchief Bandages for the Head.—These are particularly useful because of their lightness, their ease of application, and their security. (*a*) A triangular bandage is laid over the top of the head, so that the base passes straight across the forehead, the apex lying over the occiput. The ends are gathered up and carried to the back of the head, where they cross below the occipital protuberance, and then pass above the ears to the front, and are there tied. The apex is turned up over the occiput, and fixed with a safety-pin. (*b*) Esmarch describes a "four-tailed bandage" for the head as follows: "A rectangular cloth, 24 inches long, 8 inches wide, split at both ends like a split compress. To secure a dressing to the top of the head with this cloth, the two posterior ends are to be tied under the chin, and the two anterior ends under the occiput. On the other hand, to secure a dressing upon the occiput, the anterior ends are tied under the chin, and the posterior across the forehead." (*c*) A large square head cloth is also used by the same surgeon.

### Christmas Competitions.

We want none of our readers to go away for their holidays without taking some piece of work for wet days, which, when finished, can be sent to us for our Christmas parcels. So heartily were the garments for adults which we distributed last year appreciated, that we want this year to have twice the number. To encourage all to help us in this way, and to add interest to the work, we offer the following prizes, which will be awarded in books or money as the winners choose: (1) For the best pair of socks knitted by a nurse, 5s.; (2) for the best pair of socks knitted by any Hospital reader, 5s.; (3) for the best made flannel shirt, 10s.; (4) for the best made woman's blouse, 10s.; (5) for the best made flannel petticoat, 10s.; (6) for the best made and best shaped dressing gown for an invalid cut out and made by a nurse, 20s. It will be seen that No. 1 and 6 are reserved for nurses only. With regard to No. 6 we specially hope for many entries, and if we secure them we propose to give more than one prize. Flannellette is cheap, and light, and warm, and would, therefore, form the best material for the dressing gown. In judging, four marks are given for workmanship, four for shape, and two for general appearance; therefore, it is not wise to spend time on elaborate trimmings. Long seams may be done by machine.

### CONTENTMENT.—II.

We have made up our minds that we *are* discontented, that is a long step in the right direction; we will go on with braver hearts to see what the crosses are of which we complain. Some, perhaps, we have brought on ourselves, we have made it appear, possibly, that we wanted a thing to be done, and afterwards complained that it tired or annoyed us. Then we count up our grievances till we can think of nothing else, and in our very prayers complain of others rather than of ourselves. Meanwhile, we aggravate our minds by dwelling on the imaginary faults of our relations and friends, till we get quite an unreal view of their characters and actions. Does not this look like sin towards God, towards our neighbour, aye, even towards ourselves?

God has chosen our friends, our home, our pains and pleasures, that He may the more surely and swiftly perfect us and make us like unto Himself. We sin against our neighbour when we have hard thoughts of him, and against ourselves when we will not accept the blessings and comforts with which God has surrounded us. Our eyes and ears have been stopped by our discontent, we would not see the pleasant things, we would not hear the loving voices; we have sinned against Faith, Hope, and Charity. We have not trusted God, we have not hoped all things, nor believed all things, nor endured all things—in the spirit of love. But it is not too late to alter. The God of love will lead us on step by step till we are changed into His own image. Only we must be in earnest, and fixed and steadfast in our purpose. We will ask God to show us what to do, then begin with some one thing, and determine not to complain of that thing in future, and if we forget our resolution, we will humbly ask God to forgive us and help us to try again more successfully. We must go on by degrees, and as our spirits and wills grow stronger, we shall leave off murmuring and think only of those things which give us pleasure, and make us thankful.

Our hearts, which were dark and dismal, will be filled with amazement at the love and kindness which our friends show us, and which cause us to be bright and happy. We shall wonder what has given life such a different appearance and why we were formerly so blind, and our hearts so cold and lifeless.

This is true to the letter, we are painting no fancy picture, only let us try and make it our own. The Scripture says, Godliness with contentment is great riches. It is so indeed, it prevents our coveting our neighbour's goods, it makes us feel we have enough, and even to spare for those who are worse off than ourselves. Contentment is a feast, it filleth us with all good things, and it is the source of all real joy and gladness. Having gained it we shall long to express the new and overflowing love in our hearts and will cry

Oh! Lord, unlock my lips in praise,
By silence chained too long;
With fervent tune my voice shall raise
The notes of thankful song.

### A Uniform Chat.

Perhaps nothing shows more clearly the development of artistic tastes among all classes than the variety of graceful and picturesque uniforms that were noticed at the conversazione at Merchant Taylors' Hall and the presentation at Marlborough House. The eye travelled with pleasure from the simple Sister Dora cap worn by some of the private nurses to the jaunty fez of the Berry Wood attendants, and marked with relief the disappearance of the bunchy cap made on a frame, and the bibless apron, both of which were rampant a few years ago. No less remarkable is the improvement of outdoor garments. Once upon a time, when uniform was perhaps less thought of than now, our minds were often harrowed by the spectacle of a gaunt, thin nurse wound tightly in a skimpy cloak, her hair drawn back from her face till only the strained roots could be seen, and sometimes an inch of white stocking, showing above her elastic-side boots, the loops of which always stuck out back and front in a defiant way, that forced rather than attracted attention. All is vanity and mortification of spirit was written upon her brow, and in her way, she was only a few shades less objectionble, than her predecessor Sarah Gamp, a rebound from whose superfluous personality and slovenliness she was. But now we are getting the happy medium of that neatness that is not primness, and that grace and beauty of form and material that does not in the least interfere with use and suitability. It is a great advance upon the ideals of the past, that nurses have begun to understand that it is a part of their duty as ministering women to make themselves as pleasant and as bright to the eyes of their patients as possible. More and more it is beginning to be recognised that one of the great points of nursing is to draw the sufferer's mind from his aches and pains, both mental and physical. Only a few weeks ago one of the medical journals debated at length whether it would not be wise to institute a system of musical therapeutics, questioning whether sweet and soothing sounds would not afford a valuable relief from pain. Without discussing the merit of this argument, we are able to judge by experience that the mind is attracted as much through the eye as through the ear, and that the cheerful and attractive dresses of blue and pink zephyrs, of fawns, greys, soft greens, and crimsons, which are usurping the places of the eternal navy blue and black, surmounted as they are by picturesque caps and aprons, have a cheerfulness and freshness that is a continual relief to the patient. More especially does this apply to private cases where the invalid is perhaps allowed but few visitors, and is partly or wholly dependant on the nurse for companionship and change. A short time ago I was told by a medical man that in the case of one of his nervous patients, who was fast sinking into a dangerous state of lethargy from which nothing seemed to rouse her, he noticed that a gleam of interest appeared on her face as her eyes rested on the picturesque Norman cap of her new nurse. Acting on the motive that nothing is trifling when life is concerned, he got the nurse to appear in a fresh cap every day for several days, with the effect that the patient roused herself to take more and more notice; this mental stimulant being apparently the turning point in her illness.

Veils appear to be going out of fashion, and one hardly knows whether to regret them or not. Hanging in soft, free folds from the bonnet, as so few nurses know how to arrange them, they are a becoming and graceful addition to the costume, and, drawn lightly round the throat, form a most comfortable protection from east winds and draughts in railway carriages. But folded stiffly, and hanging down in the formal style that resembles nothing so much as a flattened stove-pipe, they are utterly abominable, and quite useless. It is not an unusual sight on a wet and windy day to see an unfortunate nurse with her veil straining and fluttering like a living thing, sometimes standing erect on her head with a reckless and defiant air, and again half blinding her by swishing its flabby meshes in her eyes, her hands meanwhile being occupied by her gown and umbrella. This is not an enviable state of affairs, and unless the veil is so attached to the bonnet that the corners are free to be brought to the front and fastened with a small safety-pin on windy days, it is better absent altogether. The best gauze, which is always cheapest in the long run, does not spoil by being pinned.

Nurses who are clever with their needles, as many are, will perhaps be glad to know that the round full cloak gathered on to a yoke is most easily and quickly made. Three lengths of double-width stuff are measured according to the height of the wearer, two inches being allowed for the hem. These are joined together, hemmed round the bottom and front—or the latter can be lined with a strip of contrasting colour—and sloped back and front at the top. That is to say, two inches are cut at the extreme back and front and gradually decreased towards the sides, where greater length must be allowed for the shoulders. The skirt is now ready for the yoke, which should be first cut out of brown paper and fitted into the wearer's neck till it sets on the shoulders without wrinkle or fold ; there should be no joins. When a pattern is got that fits perfectly, a cloth yoke of two thicknesses should be cut by it. The skirt is then gathered evenly into the yoke, and nothing remains to do but to put on a collar, either standing up or turning down, according to the owner's taste. It takes about five yards of material, which may be purchased in all colours at two or three shillings the yard. Any nurse with a little time on handy by following these directions, can make in a few hours a stylish and graceful cloak equal to one costing thirty-five shillings ready-made.

Let us hope that we shall long cling to our white bonnet fronts and strings—they look so fresh and dainty. I never see the cheerful faces of our nurses under the little frills without thinking of lavender and cabbage roses and all the sweet old-fashioned flowers. Perhaps the biologists would tell us that it is a remembrance of some former state when I was part of my grandmother, and went to the village church in a clean bonnet-front with posy and prayer-book in hand.

### The Royal National Pension Fund for Nurses.

It is gratifying to find that not only at home are the advantages of the Pension Fund appreciated. A nurse from Gibraltar writes this week : "I am No. 169 on the roll of the Royal National Pension Fund, and my premium never becomes due without my blessing the day I joined, and thinking what an excellent thing it is for us all. Every nurse, I am sure, is most grateful to all those who have so kindly and generously helped to make the Fund what it is to-day. My young sister last year became a probationer. I got her to join at once, and I feel quite happy on her account, now I know she has made a beginning. I might write pages, but it would all amount to the same thing, I feel so truly grateful for all that has been done for us."

[No. 169 is perfectly right in the views she expresses. Every one of the first two thousand nurses should make up her mind, now she is herself secure, to set to work, and to provide that at least one other nurse may place her savings in the Fund forthwith. By such a proceeding nurses would benefit not only themselves but their sister workers throughout the country.—Ed.]

### The Princess of Wales' Fund for Mrs. Grimwood.

The following sums are acknowledged in connection with this fund, the list for which is now closed :—Nurses, Lavinia Tidy, 2s. ; E. A. Heeton, 1s. ; T. E. M. Hart, 1s. ; E. Turner, 1s. ; Robins, 1s. ; M. Wright (Matron), 2s. ; W. W., 1s. ; From Two Nurses, 2s. 2d. ; Cassaidy, 2s. ; Sister Garriock, 2s. 6d. ; Nurse Flora Kirwood, 5s. ; Sister L. C. Fisher, 2s. *Erratum.*—Nurse Garbing was entered as Garling.

### The Nurses' Bookshelf.

### A SERIES OF TEXT BOOKS.*

The first volume of the Nursing Record Series of Text Books has, for some reason, not been sent us, but three volumes of varying price and varying worth are to hand. First, there is "Norris's Nursing Notes"—an old friend in a new form. Originally published by Miss Williams and Miss Fisher in 1877, these "Notes" reappeared lately in the pages of the *Record,* and now, revised by Mrs. Norris (*née* Williams), they appear in book form again. They make a very useful volume, and are considerably improved by the introduction of diagrams, a glossary, and a table of weights and measures. The tone of the book is good throughout ; the knowledge is that common to all nursing hand-books, and has no distinctive character save for the valuable addition of the glossary. There is, however, a short and simple chapter on "Obstetric Nursing," and one on "Modes of Death," which are serviceable, and contain information which some manuals lack. "Practical Electro-Therapeutics," by Dr. Harries and Mr. Newman Lawrence, is a very concise volume, well illustrated, and furnished with a full index. The authors plunge straight into their subject and stick to business throughout, and they have the advantage of treating a subject on which few cheap hand-books have been written. The diagrams and plates are excellent and there is a glossary and full index at the end.

Of the third volume of the series we cannot speak so highly ; it is good so far as it goes, but it is a terribly small shillingsworth. There are only sixteen pages of matter, and the rest of this slim book consists of advertisements and title pages. It has certainly no right to be called either a "manual" or a "text-book" of massage, for any masseuse seeking technical information here would infallibly be disappointed. We recommend that these few pages be used as an introduction to a proper text-book on massage, for as "hints" there is something to be learnt from them.

### BETWIXT TWO LOVERS.†

We thought Mrs. Jarley, of celebrated waxwork memory, was dead, and lo ! she appears before us resuscitated on the platform of literature. She has taken a new form, and the mantle of the prophetess has descended on the shoulders of one of the opposite sex. The garment has fallen, but in the fall some of its virtue has scattered to the four winds. Our old friend in petticoats never failed to amuse us. Colonel Hamilton ignores this part of the programme entirely. He places his puppets in a row before our eyes, provides us with a microscopic description of each, both mental and physical, then pulls the string, but when the figure moves, it chooses its own line of action, as if no hand were there to guide it. We feel quite sorry for Colonel Hamilton, with the tribe of unruly spirits which he has evoked, and failed to master. But the plotters failing, we turn to the plot. We look to the consummation of the title. Alas ! we fail to find one solitary lover, much less two such beings, properly so called. Our study of the heroine is not much more successful. According to the author, she is as "Diana whom the Ephesians worshipped." According to us, she is a very commonplace young woman, with but one attribute in common with the goddess, and that is an aptitude to condone infringements of our moral codes, and this is truly more Olympian than English as yet. Nevertheless it is very convenient for No. 2 of the so-called lovers, who, first choosing to make himself acquainted with the heroine's feeling of affection for him (which for our part we thought she did not trouble to disguise), and runs off with a married woman by way of change. This proceeding is placed before us as if it were a matter-of-course every-day occurrence. We fully believe that Colonel Hamilton's principles are better than the impression of them which he conveys to his readers. The situations he presents are, however, fortunately not sufficiently interesting to be dangerous. There is more poetry of description in the earlier part of the book, where the author dwells much upon sunsets, and where he has made use of a very florid collection of terms. He we learn

* "Norris's Nursing Notes" (2s.); "Practical Electro-Therapeutics" (1s. 6d.); and "Massage for Beginners" (1s.). Sampson Low and Co.

† "Betwixt Two Lovers." By Colonel Rowan Hamilton. Two vols. London : F. V. White and Co.

that gigantic yews in the "winter Solstice render the barren landscape joyous with the perennial verdure of their clothing." Much care has been also lavished on descriptions of breakfast tables, and sporting scenes. They are all treated very much in the same manner. We cannot help thinking that a little more attention to dramatic situations and less to description of details would tend to enhance Colonel Hamilton's success as an author.

### The Princess of Wales and the Nurses.

Photographs are pouring in for the screen which the First and Second Thousand nurses have decided to offer for the acceptance of the Princess of Wales on her next birthday. As not more than 1,000 photographs can be placed on the screen those nurses who wish to show their appreciation of the Princess's kindness to them should send carte and postal order for 2s. immediately, to Screen, care of Miss Pritchard, The Lodge, Porchester Square, London, W., or they may be too late. The following are postal orders acknowledged: From Nurses L. Wilshaw, E. Barkwith, Still, E. Burton, Lucie, A. Bridle, S. Openshaw, Speat, A. Speat, A. Macvicar, J. Hodgetts, E. Skelton, S. Richardson, A. Galbraith, A. Rays, C. Porter, L. Weatherby, C. Dearth, C. Cox, Ellis, R. Johnson, S. Philips, E. Stanton, M. Guernsey, G. Redit, A. Faulder, Berrywood, C. Walton, F. Grigg, A. Spring, S. Steer, F. Kennett, L. Barnett, E. Beswick, M. Pitcher, C. Meany, J. Greene, A. Moorhead, S. Buckland, C. Missenden, A. Missenden, Fanny Daniel, Janet Jones, Annie Hughes, Mary Evans, M. Wray, J. E. Jones, E. Missenden, J. Lloyd, A. Kelway, E. Brigstock, M. J. Garrett, M. Holloway, A. Laurie, H. Stockwell, E. Butler, Summers, A. Fulter, M. Lawrence, A. Hagnes, T. Steer, J. Tuder, L. Mason, F. Lewis, E. Leake, E. Lempriere, F. Kidd, C. Stevens, M. Cross, M. Wallis, T. Steer, Alfard, Wilson, E. Chapman, F. Hargreaves, P. Sandall, E. Jacob, E. Nichols, P. Munton, V. Charridy, Atkins, L. Carter, S. Doughty, Brown, J. Grant, H. Geary, A. Brierley, M. Harris, H. Harnden, E. Farrar, M. Hill, E. Cockagne, M. Elsby, M. Oliver, A. Espley, S. Butter, Scott Palmer, E. Hosgood, M. A. Tomkins, F. Storm, F. Heynes, M. C. M. Trabs, M. Emes, M. Dinsdale, M. Burdett, A. Croydon, J. Goldsmith, K. V. Macintyre, S. E. Wempson, E. Ryan, A. Cole, F. Smith, J. L. Anderson, N. Manton, L. Wills, K. Kent, E. A. Larkum, E. A. Waters, E. M. Peter, E. S. Mowers, I. Bothams, S. J. Lilley, E. Francis, E. Clay, J. M. Thurston, S. Baxter, B. Chapman, L. J. Groves, Cowdell, A. Griffith, S. Watson, A. Latter, A. Newport, A. Thorpe, E. A. Mackaness, M. A. Bliroom, H. E. Elliott, M. McCartney, F. S. Saunders, H. Cole, C. Barton, E. Langley, E. Bellamy, M. E. Macdonnell, L. Robbins, E. A. Smirly, L. A. Bacon, S. Walker, E. North, J. Porley, F. Shaw, F. Stockwell, S. Bellamy, C. Hutchinson, M. Calver, E. Gates, E. Whitehead, M. Edmonds, P. Kirk, Baskin, J. Solby, E. Mills, Batchelor, A. Woods, M. Arnold, S. Edwards, and A. Winter.

### Derby Children's Hospital.

On the 12th inst., the Lady Superintendent took the nurses at the Derby Children's Hospital for a picnic to Alton Towers. A beautiful avenue about a mile long leads to the Towers and grounds, and under the shade of these trees the nurses partook of the good fare provided in the luncheon basket. A visit was afterwards paid to the gardens, conservatories, Swiss Cottage, and the Flag Tower, from whence there was a delicious breeze, and very extensive view of the neighbourhood round. A drive and a walk through the pretty village of Alton, passing through the churchyard, and tea, took up the remainder of the day, and soon after seven the return journey was commenced. All the nurses seemed very happy and thankful for what we believe proved one of the happiest days they had ever spent.

### Appointments.

Great Yarmouth Hospital.—The Matron appointed in succession to Miss Wiltshire, is Miss C. Bowman, who was trained at County Hospital, York. She was subsequently Head Nurse for about two years in the Great Yarmouth Hospital, and finally Night Superintendent at the Royal Glasgow Infirmary.

Cork County Hospital.—Miss M. Ward Roberts has been appointed Matron of the County Hospital and Southern Infirmary, Cork, in place of Miss Franklin who has resigned, after having filled the post for forty years. Miss Roberts received her training at St. George's Hospital, London, where she was for some time, and has had experience in nursing with hospital management and district work, and has been for some little time working at the Royal Berks Hospital, Reading.

Jaffray Hospital.—Miss Harriet M. Pidgeon has been appointed Matron to this institution. Miss Pidgeon worked at Great Ormond Street from 1875 to 1881, and afterwards at the Leicester Infirmary, at Scarborough, and at Nice. For the last four and a-half years she has had charge of the Cottage Hospital at Hammerwich, and her testimonials show how capable and competent an administrator she proved herself to be. The Chairman of the Hammerwich Hospital, indeed, declares that its success is mainly due to her management.

### Notes and Queries.

### Queries.

(30) I am Matron of a small provincial. Little is to be saved, as you well know, by any of us, and what little I have scraped together is already invested, and it would serve (so far as I can see) no purpose to take it out, and reinvest it in the National Provident Fund. Now, how am I to obtain a pension? A long term of years in one place does not appear to me to bring forth any gratitude from committees or the general public; and after one's working days are over and one is no more use, one certainly can go out into the cold. We old Matrons and Sisters have no chances to help ourselves as the young ones have. Hence these few remarks.—*Annie Schofield.*

(31) I am anxious to hear of a free convalescent home for a girl recovering from hysteria, and generally out of health.—*J. Temple.*

(32) *A Matron* will be glad to know what hospitals give retiring pensions to their nurses. What is the necessary age, and what the amount given?

(33) *Ambulance Classes.*—Would any readers of The Hospital kindly tell me the best way to get an ambulance class formed here? There are plenty who would gladly join it.—*An Exmouth Reader.*

### Answers.

*Water Beds.*—I am surprised at the want of knowledge displayed by your correspondents re the filling of water beds, none of whom seem to know that it is necessary to add a few quarts of one in twenty carbolic to the water. If one of my probationers were to fill a water bed without adding the carbolic, I should be astonished at her ignorance and want of sense. Judge, therefore, of my surprise at the remark that the nurse would "find it rather smelly." The patients in a ward would (to put it mildly) think it rather unpleasant if a water bed in which no carbolic had been put were to be emptied in their presence after being in use three months. It is by such careless acts of ignorance that typhoid, &c., finds its way into our hospitals and homes.—*Sister.*

*Holidays.*—Nurse A. advises nurses who wish to spend their holidays at the Isle of Wight to take up their temporary quarters in Newport. The air on the hills is bracing, and the view splendid. All home comforts to be had while with Mrs Short, 7, Union Street.

*Miniature Charts.*—As stated, Nurse Beavis can obtain these from H. K. Lewis, whose shop University College nurses must know, at any rate.

(26).—Having had much experience with young babies, I can thoroughly recommend the following books on the subject: "Advice to a Mother on the Management of her Children," by Pye Chavasse, 2s. 6d.; "Our Baby," by Mrs. Langton Hewer, 1s. 6d., publisher, John Wright and Co., Bristol; "Babies, and how to take care of them," price 1s., Ward, Lock, and Co. (excellent for nurses and young mothers); "What Every Mother should Know," by E. Ellis, M.D., 1s. 6d.; "Our Babies, and how to take care of them," by Florence Stacpoole. L.O.S., price 3d., 70 pages, publisher, A. Gardner, Paternoster Row.—*Nurse Gertrude.*

(28).—Medical lady will be glad to correspond in answer to query. Fee, 2s. 6d. for advice by correspondence per letter.—*Sister*, 132, Christchurch Road, Boscombe, Hants.

(29) The name of the convalescent home near Liverpool required by a reader of The Hospital is, I expect, the Woolton Convalescent Home, which I know is a few miles from Liverpool.—*B. Chapman.*

(30) In answer to the inquiries made by the Matron of the Torbay Hospital, we beg to state that any one putting their money into the Royal National Pension Fund receives 2½ per cent. compound interest, and after two years, can withdraw their investments at will. In addition they receive a share of the profits, and also of the Donation Bonus Fund—all the advantages make the percentage received much higher than can be obtained elsewhere. The money can be paid in irregularly on deposit at 2½ per cent., and when the sums reach £10 they will be converted into a policy earning the larger interest. Finally, those who join become eligible for a grant from the Morgan Benevolent Fund, if incapacitated. We should be glad to know wherein the greater difficulty in the Matron's or Sister's saving lies. It seems to us that their greater experience increases their responsibility to save.

### The Other Benny.

"What a curious thing to be sure! Do you mean to tell me that both of these little chaps are called Martin, and are both broken leg cases?" and the visiting doctor stared down at the new occupants of two small beds in the ward.

"Yes, and funnier still," said Nurse Dorothy, with irrepressible smiles breaking over her face, "they each seem to have been christened 'Benny,' and," she added more gravely, "each is 'the only son of his mother, and she is a widow.'"

"But mother's had plenty aside me," spoke up one of newcomers jealous for that mother's credit; "heaps, but they're all dead now, 'cept me; I'm livin'."

"Well, yes," observed the doctor musingly, as he eyed the white-faced morsel of humanity who had just spoken carefully all over. "You are, at present," he murmured, "but if we don't do our best you'll slip away. However, we'll see! I've pulled through worse than you. And" he went on aloud, "what about the other Martin; are you living too, eh?"

"Yes, sir, oh, yes!" was the quiet answer. "I've got to, you see, sir, cos mother, she depends on me."

The other Benny was smaller, paler, even thinner than the first, and the doctor looked longest at him while Nurse Dorothy explained the circumstances of their arrival the night before, within an hour of each other, in the accident ward. It was one of those rare, but still genuine, occurrences proving that truth can keep well up alongside of fiction. The two Martins, human scraps of the great metropolis, of the same name, but hitherto unknown to each other, had been knocked down; the one in front of a brewer's dray, a wheel of which had crunched over his leg; the other, below a falling ladder; and both had been carried into the same hospital. The curious similarity of the cases—duplicates, so to say—awoke a wonderful interest among doctors and nurses. When the two limbs were set and bandaged, and their small proprietors made comfortable in their respective cots, everybody in the hospital made excuses to pay them flying visits. Other patients who were bedridden craned their necks to get a peep at the newcomers, and, for a little thing makes an excitement in the calm, orderly tenour of hospital life, the two Martins were the heroes of the moment.

As for the little lads themselves, they speedily became fast friends, and took such a neighbourly interest in the welfare of each other's broken limb that both lost sight of a good deal of their own individual sufferings.

"A good practical lesson for those willing to learn," declared pleasant-faced Nurse Dorothy. "And," she went on, turning to her charges, "I've such a fine idea in my head. Do you two Bennies like music?"

"Oh, yes'm!" eagerly said the frailest of the two boys, the one who began, unaccountably, to be known as the other Benny. "I can play ''Ome, Sweet 'Ome' on the penny whistle."

"You can? Well, you shall each have a little flute and play duets," and Nurse Dorothy did not forget her promise.

After that being in hospital was simple bliss. All day long the two Bennies lay on their beds, in their red flannel jackets, untiringly tootling the one unvarying tune. But the other Benny did not mend; there was no disguising that fact. He grew shadowy while his small chum made way surprisingly fast. The two widow-mothers came and crooned over the little men time after time, and while one woman went off rejoicing the other carried away a heart that grew heavier after each visit.

"It's no use," the doctors told Nurse Dorothy, "you'll soon lose them both. The one will go out sound; it's a first-rate cure, that leg; the other will sink rapidly, he's got no constitution to work upon."

But still the two Martins, unconscious of the fiat that had gone forth, played their one tune unflaggingly; only each day the other Benny began to take a longer rest between. His flute grew heavier and heavier to hold, he could not make out why. At last there came days when only one Benny tootled "Home, Sweet Home," while the other Benny listened with folded hands and a blissful, contented smile.

"Quick, Benny, quick! Let's play the tune before I go home:" a sharp, thin voice broke on the midnight silence, and both the Martins fumbled under their pillows for the beloved flutes.

On the astonished ears of the suddenly aroused patients stole the old familiar air; for a few bars it was a duet; then it finished as a solo, for the other Benny had gone—as the doctors predicted he would—to the sweetest of all sweet homes.

Both the beds are now empty, for hale and hearty little Benny Martin has taken away his leg quite cured, and in his home he never tires entertaining his mother with long accounts of the best holiday he ever had in his life—the spell in the great, clean, wholesome hospital which was to him a veritable Palace of Delight.

### Wants and Workers.

[Under this heading, we propose to try whether we can be useful to our readers in making the wants of some known to others who are willing to do what work they can to aid the great cause of curing and cheering the sick. Wants can only be inserted from those who are connected with some institution or association, or who are willing to have their full name and address printed.]

*Lady Superintendent*, King's Lynn Hospital, begs to acknowledge gratefully number of beautiful Christmas cards from Anon. Post mark, Euston Road. Will not others help?

### Amusements and Relaxation.

### SPECIAL NOTICE TO CORRESPONDENTS.

**Third Quarterly Word Competition commenced July 4th, 1891, ends September 26th, 1891.**

Competitors can enter for *all* quarterly competitions, but no competitor can take more than *one first prize* or *two* prizes of any kind during the year.

Proper names, abbreviations, foreign words, words of less than four letters, and repetitions are barred; plurals, and past and present participles of verbs, are allowed. Nuttall's *Standard* dictionary only to be used.

N.B.—Word dissections must be sent in WEEKLY not later than the first post on Thursday to the *Prize Editor*, 140, Strand, W.C., arranged alphabetically, with correct total affixed.

The word for dissection for this, the EIGHTH week of the quarter, being

"SNIPE."

| Names. | Aug. 13th. | Totals. | Names. | Aug. 13th. | Totals. |
|---|---|---|---|---|---|
| Paignton | 31 | 237 | Christie | — | 44 |
| Psyche | 34 | 237 | Dulcamara | 33 | 230 |
| Hope | — | 47 | Nurse J. S. | 32 | 237 |
| Lightowlers | 34 | 249 | Qu'appelle | 33 | 241 |
| Wizard | 10 | 175 | E. M. S. | — | 68 |
| Wyameris | — | 46 | Jenny Wren | 32 | 220 |
| Dove | — | 46 | Carpe-diem | — | 65 |
| Punch | — | 181 | Grannie | — | 36 |
| Ivanhoe | — | — | Nurse G. P. | 25 | 1[illegible]3 |
| Tinie | — | 93 | Goodnight | 21 | 122 |
| Agamemnon | 34 | 246 | Gamp | — | — |
| Nurse Ellen | — | 86 | Charity | 19 | 104 |

### Notice to Correspondents.

All letters referring to this page which do not arrive at **140, Strand, London, W.C.**, by the *first post on Thursdays*, and are not addressed PRIZE EDITOR will in future be disqualified and disregarded.

N.B.—Each paper must be signed by the author with his or her real name and address. A *nom de plume* may be added if the writer does not desire to be referred to by us by his real name. In the case of all prize-winners, however, the real name and address will be published.

## Figures and Tables

**Figure f1:**